# Cloning and functional validation of early inducible *Magnaporthe oryzae* responsive *CYP76M7* promoter from rice

**DOI:** 10.3389/fpls.2015.00371

**Published:** 2015-05-22

**Authors:** Joshitha Vijayan, B. N. Devanna, Nagendra K. Singh, Tilak R. Sharma

**Affiliations:** National Research Centre on Plant Biotechnology New Delhi, India

**Keywords:** *Arabidopsis*, *CYP76M7*, GUS, *Magnaporthe*, promoter analysis, rice

## Abstract

Cloning and functional characterization of plant pathogen inducible promoters is of great significance for their use in the effective management of plant diseases. The rice gene *CYP76M7* was up regulated at 24, 48, and 72 hours post inoculation (hpi) with two isolates of *Magnaporthe oryzae* Mo-ei-11 and Mo-ni-25. In this study, the promoter of *CYP76M7* gene was cloned from rice cultivar HR-12, characterized and functionally validated. The Transcription Start Site of *CYP76M7* was mapped at 45 bases upstream of the initiation codon. To functionally validate the promoter, 5′ deletion analysis of the promoter sequences was performed and the deletion fragments fused with the β*-glucuronidase (GUS)* reporter gene were used for generating stable transgenic *Arabidopsis* plants as well as for transient expression in rice. The spatial and temporal expression pattern of GUS in transgenic *Arabidopsis* plants and also in transiently expressed rice leaves revealed that the promoter of *CYP76M7* gene was induced by *M. oryzae*. The induction of *CYP76M7* promoter was observed at 24 hpi with *M. oryzae*. We report that, sequences spanning -222 bp to -520 bp, with the cluster of three W-boxes, two ASF1 motifs and a single GT-1 element may contribute to the *M. oryzae* inducible nature of *CYP76M7* promoter. The promoter characterized in this study would be an ideal candidate for the overexpression of defense genes in rice for developing durable blast resistance rice lines.

## Introduction

Plants are being exposed to various biotic and abiotic stresses and they have developed smart developmental and defense mechanisms to counteract these stress conditions. Among biotic stresses, plant pathogens cause widespread damage to the crop plants. The growth and development of organisms is a complex network of different gene products which are controlled by upstream regulatory elements of genes or promoters which induce spatial and temporal specificity to the expression pattern of respective genes. Rice blast disease caused by *Magnaporthe oryzae*, a hemibiotrophic filamentous plant pathogenic fungus, is one of the most dreaded diseases of rice crop causing an average annual yield loss of 30–50% in rice growing regions of the world (Sharma et al., 2012).

The *R-* gene mediated host plant resistance is considered to be one of the most effective, economically feasible and environmental friendly approaches for the effective management of rice blast disease. However, host resistance is short lived due to highly variable nature of *M. oryzae*. Therefore, use of multiple *R*- genes is considered as the most viable and durable option for management of rice blast disease (Hulbert et al., 2001; Das et al., 2012; RoyChowdhury et al., 2012; Devanna et al., 2014). An initial strategy adopted for achieving this was to overexpress resistance genes under the control of constitutive promoters, but in many instances the improved disease resistance was accompanied by reducing growth and associated yield penalty in commercial crops (Hammond-Kosack and Parker, 2003). Therefore the problems associated with constitutive expression can be overcome by using spatially and temporally inducible promoters. There are a number of cloned and characterized promoters which show constitutive, inducible (as pathogen, wound, and chemically induced) and tissue-specific (such as root, stem, pollen, and green tissue-specific) expression (Gurr and Rushton, 2005). Identification and characterization of pathogen inducible promoters would be of larger practical value as they avoid the unnecessary physiological burdens associated with constitutive expression of transgenes on the host plant by restricting their expression to specific time and the site of infection (De Wit, 1992). An ideal pathogen-inducible promoter is the one which is rapidly activated in response to a wide range of invading pathogens and therefore remain effective in providing early and broad-spectrum resistance to the pathogens. In view of this, it is of utmost importance to isolate and characterize a pathogen inducible promoter for the efficient regulation of transgenes and to provide broad spectrum resistance against invading plant pathogens. There are a large number of known pathogen-inducible genes in plants and promoters of a few of those genes have been characterized (Rushton and Somssich, 1998; Gurr and Rushton, 2005). Among the well characterized pathogen inducible promoters, two groups of *cis*-regulatory elements like GCC-like elements (Ohme-Tagaki et al., 2000) and the W boxes (Rushton et al., 1996; Eulgem et al., 2000) have been widely studied and functionally validated. Secondary metabolites produced during biotic and abiotic stresses are reported to be involved in plant resistance mechanism and therefore the promoters regulating the expression of these genes coding for secondary metabolites are the ideal candidates for the cloning and characterization of novel pathogen inducible promoters.

The *CYP76M7* gene (LOC_Os02g36110), a member of P450 monooxygenase, has been reported to be induced after chitin elicitor treatment (Okada et al., 2007). Further, it is known to have a role in the production of antifungal phytocassanes and belongs to a diterpenoid biosynthetic gene cluster located on the rice chromosome 2 (Swaminathan et al., 2009). Previously, phytocassanes have been shown to accumulate in rice during *M. oryzae* and *Rhizoctonia solani* infection (Koga et al., 1995). Our microarray based expression study and real time PCR analysis have also confirmed that *CYP76M7* gene was highly induced starting from 24 h after challenged with *M. oryzae* isolate Mo-ei-11 and Mo-ei-25 and was consistently up regulated at both 48 and 72 hours post inoculation (hpi; Vijayan, unpublished data). Recently, in another study, which used RNA sequencing approach for transcriptome analysis at 24 hpi also reported a high level induction of *CYP76M7* gene during an incompatible interaction (Bagnaresi et al., 2012). However, the promoter region of this gene has not been characterized at the molecular level. The objectives of the present study were to isolate, characterize and functionally validate a promoter induced by the rice blast fungus *M. oryzae*.

## Materials and Methods

### Plants, Fungal Strains, and Oligos

*Magnaporthe oryzae* isolates Mo-ni-25 (Dehradun, Uttarakhand, India) and Mo-ei-11 (Hazaribagh, Jharkhand, India) was collected from the respective locations in India. Seeds of *Arabidopsis thaliana* L. (Col-0) and indica rice cv. HR-12, susceptible to both *Magnaporthe* isolates was available at the institute. HR12 was used in the study because the initial microarray experiments, which indicated the early expression of *CYP76M7,* were performed using rice line HR-12 (Vijayan, unpublished data). The list of oligos used in the present study is given in the **Table 1**.

**Table 1:** List of oligos used in the present study.

Primer name	Primer sequence (5′–3′)
Pcyp2004F	ACAAGCTTAGATCTATGGTTTGTAGGTTTTT
Pcyp2004R	ACGGATCCGTTCTTTTTCTCTGGTTCTACCTG
DELPcyp1456F	ATAAGCTTTGTGTGATGAGCGTCCTTCC
DELPcyp1167F	ATAAGCTTTCGGTCGAACACGCATAGC
DELPcyp520F	ATAAGCTTCAGCCGTGAGAATCCGTATC
DELPcyp222F	ATAAGCTTCGCCATGCAGTAAGGGTATATTC
NPTIIF	TGAATGAACTGCATGACGAG
NPTIIR	AGCCAACGCTATGTCCTGAT
Pcyp GSP1	GGGATGAACACCATGG
Pcyp GSP2	CGCACGCGCGGAAGGTGTC
Pcyp GSP3	AGGTGGCGGTCGTACTTGG
PcypREALF1	CCAAGTACGACCGCCACCT
PcypREALR1	GCTCGGGATGAACACCATGG
18SRNA-F	CTACGTCCCTGCCCTTTGTACA
18SRNA-R	ACACTTCACCGGACCATTCAA

### Inoculation with *M. oryzae*

Seedlings of susceptible rice cultivar HR-12 were grown under standard physiological conditions of 16 h light and 8 h dark photoperiod at 25 ± 2∘C. Two-weeks-old rice seedlings were inoculated with two highly virulent and geographical diverse isolates of *M. oryzae* Mo-ni-25 and Mo-ei-11. Conidia of *M. oryzae* isolates were collected from the culture grown on oatmeal agar plates by washing with 0.25% gelatin and conidial concentration was adjusted to 10^5^ spores ml^-1^ using a haemocytometer. Inoculum was sprayed with an atomizer to create fine droplets of spore suspension, which are retained on the plants. For mock control, plants were inoculated with 0.25% gelatin only. The experiment was carried out under controlled growth conditions at 25 ± 2∘C and 90% relative humidity.

### Total RNA Extraction and cDNA Synthesis

In order to get better results, total RNA was extracted from three different biological replicates of *M. oryzae* and mock inoculated leaf tissues of rice using the Spectrum Plant Total RNA Kit (Sigma). The isolated total RNA was quantified by using Nanodrop quantifier. From each sample, 5000 ng of DNase treated total RNA was used as template for first strand cDNA synthesis. cDNA synthesis was carried out using Protoscript M-MuLV First Strand cDNA Synthesis Kit (Cat. No: E6500S, NEB) according to the manufacturer’s instructions.

### Candidate Promoter Cloning and Quantitative Gene Expression Analysis

The candidate *CYP76M7* gene was selected based on in-house generated microarray experiments data (Vijayan, unpublished data). Further, RT-qPCR was performed to study the expression analysis of *CYP76M7* gene (LOC_Os02g36110) using exon specific primers PcypREALF1 and PcypREALR1 (**Table 1**). The primers were designed using QuantPrime software^1^. cDNA mixture of 2 μl was used as a template from each sample. The reaction mixture 20 μl was prepared according to the manufacturer’s protocol (KAPA Biosystems USA). 18S rRNA primers were used as internal control and PCR was run using Light Cycler 480 II PCR system (Roche Diagnostics, Germany). Each sample was taken as triplicates under following PCR conditions: initial DNA denaturation at 95∘C for 3 min followed by 45 cycles of amplification (denaturation at 95∘C for 3 s; primer annealing at 60∘C for 20 s and primer extension at 60∘C for 1 s). The data obtained was normalized with the values of rice 18S gene using the ΔΔC(T) method (Livak and Schmittgen, 2001).

### Pathogenesis Study of *M. oryzae* on *Arabidopsis*

In earlier studies it has been shown that *M. oryzae* causes typical infection on *Arabidopsis* (Maeda et al., 2009, 2010; Park et al., 2009; Nakao et al., 2011). Hence, for the present study we used *A. thaliana* L. (Col-0) for genetic transformation and functional characterization of *CYP76M7* promoter. Sterilized seeds were sown on Murashige and Skoog (MS; Murashige and Skoog, 1962) medium supplemented with 0.8% agar and 440 mg/l CaCl_2_. Plated seeds were kept in the dark for 2–3 days at 4∘C for stratification and subsequently transferred to light and dark regime (16 h light and 8 h dark) at 22∘C under 150 μmol m^-2^ s^-1^ photon flux density and 65% relative humidity. Four-leaf stage seedlings were later transferred to solarite filled pots and further grown at 22 ± 2∘C under long-day conditions in the National Phytotron Facility (NPF). The virulent *M. oryzae* spore suspension (at a concentration of 1 × 10^5^ spores/ml) was used for both spray and spot inoculation of fully expanded healthy rice leaves in three replicates. Both mock and pathogen inoculated plants were grown at 22 ± 2∘C and 90% relative humidity in dark for initial 24 h and later in light and dark regime of 16 and 8 h, respectively. Phenotypic observations were recorded 3 days post inoculation (dpi).

### 5′ RACE for Mapping TSS

The Transcription Start Site (TSS) of *CYP76M7* transcript was mapped by 5′ RACE (Rapid Amplification of cDNA End) using 5′ First Choice RLM-RACE kit (Ambion, USA). The total RNA isolated from the leaf tissues of pathogen challenged rice plants as described earlier was used as a template for 5′ RACE PCR. RACE PCR was performed according to the manufacturer’s protocol. The first strand cDNA synthesis was performed by using GSP1 primer specific to *CYP76M7* exon, and in the subsequent steps of PCR based amplification, gene specific primers GSP2 and GSP3 designed from the region upstream to GSP1 were used (**Table 1**).

### Cloning and Analysis of the *CYP76M7* Promoter Sequence

Promoter region of approximately 2.0 kb was PCR amplified using Pcyp2004F and Pcyp2004R primers specific to *CYP76M7* (**Table 1**) and cloned in pGEM-T Easy cloning vector (Promega Corporation, USA) following the manufacturer’s instructions. The clones were confirmed by PCR and restriction enzyme digestion. Three positive clones were custom sequenced (Xcelris Labs Ltd., India) using Sanger’s dideoxy method. Each fragment was sequenced twice from both the ends using universal SP6 and T7 specific primers. The sequence reads so obtained were further assembled using Phred/Pharp/Consed Software Package^2^ (Ewing and Green, 1998). The high quality promoter sequence (>30 Phred) assembled was compared using NCBI BLAST with the promoter sequence of the candidate *CYP76M7* gene, which was retrieved from the NCBI database using LOC_Os02g36110 sequence as query. The sequence (P0025F02) showing 100% match with our query sequence was selected and used for *in silico* analysis. Gene prediction was performed using online gene prediction tool FGENESH software^3^. For *in silico* analysis of *cis*-regulatory elements in the 2.0 kb promoter region upstream to the defined TSS we used online software NEW PLACE^4^ (Higo et al., 1999).

### Development of *CYP76M7* Promoter Deletions and GUS Reporter Gene Fusion Constructs

High quality consensus *CYP76M7* promoter sequence obtained in this study was used for the development of promoter-GUS fusion constructs in plant expression binary vector pBI101, with reporter gene β*-glucuronidase* (*GUS)*. For functional validation of the *CYP76M7* promoter, 5′ end deletions of the promoter regions were obtained by PCR amplified fragments. The five sets of forward primers (Pcyp2004F, DELPcyp1456F, DELPcyp1167F, DELPcyp520F, and DELPcyp222F) used for PCR had 5′ *Hin*dIII restriction enzyme site and were specific to 2030, 1482, 1193, 546, and 248 bp positions upstream of the TSS of *CYP76M7*. The common reverse primer (Pcyp2004R) carrying *Bam*HI restriction enzyme site was designed from 5′ UTR region (+25 bp; **Table 1**). Each PCR amplified and purified fragment was digested with *Hin*dIII and *Bam*HI restriction enzymes (New England Biolabs, UK). Digested products were ligated into the *Hin*dIII and *Bam*HI pre-digested pBI101 vector to create promoter::GUS constructs (Supplementary Figure S1). The recombinant clones were confirmed by PCR, restriction digestion analysis using electrophoresis on 1% agarose gel and sequencing.

### *In planta Agrobacterium* Mediated Transformation of *Arabidopsis* Plants

*Arabidopsis thaliana* (Col-01) plants were grown for 4 weeks at 22 ± 2∘C temperature, 18 h day and 6 h night conditions at the NPF, New Delhi. Four-weeks-old plants having maximum unopened buds were used for floral dip transformation of different *CYP76M7* deletion constructs. This was done by inverting the pot and dipping the unopened buds and leaves of the plants into *Agrobacterium* suspension prepared in 5% sucrose with 0.05% Silwet-L77 solution. The transformed plants were kept at 12 h in dark and thereafter washed with distilled water. Plants subjected to transformation were then grown to maturity, and then seeds harvested from these plants were sterilized and subjected to selection on kanamycin (50 μg/ml) containing MS medium as described in earlier sections. Overall transformation efficiency of *Arabidopsis* plants with the different CYP76M7 deletion constructs was found to be between 0.32 and 1.05% (**Table 2**). The putative transgenic *Arabidopsis* plants which grew to four-leaf stage were transferred from the antibiotic containing media plates to solarite containing pots and further grown to maturity to obtain T_2_ seeds. Transgenic *Arabidopsis* lines which showed 3: 1 Mendelian segregation ratio was utilized for further experiments (**Table 3**). Six transgenic homozygous lines each from all the *CYP76M7* promoter deletion construct as well as constitutively expressing CaMV 35 S promoter were selected for functional validation at T_2_ generation. Transgenic T_2_ plants were also confirmed for the presence of *CYP76M7* promoter and *NPTII* sequence by PCR based screening. *NPTII* gene forward primer and *CYP76M7* reverse primer were used for PCR amplification and validation.

**Table 2:** Transformation efficiency of *Arabidopsis* plants using floral dip method.

Promoter Construct No. (bp)	Total plants screened^1^	No. of positive plants obtained	% transformation
Pcyp2004	2836	14	0.45
Pcyp1456	1237	6	0.48
Pcyp1167	2273	24	1.05
Pcyp520	1438	5	0.34
Pcyp222	1832	6	0.32

*^1^T1 seeds of transgenic Arabidopsis were screened on MS medium containing kanamycin antibiotic*.

**Table 3:** The segregation ratio of Knr:Kns populations of transgenic plants.

Transgenic line	Green seedling^∗^	Yellow seedling^∗^	Green/yellow seedling^∗^
(1)	78	25	3.12
(2)	132	53	2.49
(3)	113	40	2.82
(4)	98	28	3.06
(5)	81	32	2.53
(6)	124	38	3.26

*^∗^Selection on media with kanamycin (50 mg/ml), the resistant seedling (Knr) are green while the sensitive (Kns) are pale-yellow; the ratio of green/yellow seedling represents the inheritable character of transgenes*.

### GUS Histochemical Assay for *M. oryzae* Responsiveness

Four-weeks-old healthy and uniformly grown transgenic and control *Arabidopsis* seedlings were challenged with highly virulent isolates (Moei-11and Mo-ni-25) of *M. oryzae*. Conidia of *M. oryzae* isolates were collected from cultures grown on oatmeal agar plates by washing with 0.25% gelatin, with the conidial concentration adjusted to 10^5^ spores ml^-1^. Plants were spot inoculated with conidia suspension on different parts like leaves, stem and buds. For mock control, plants were inoculated with 0.25% gelatin only. The experiment was carried out under controlled growth conditions at 22∘C and 90% relative humidity. Samples were collected at 24 hpi from inoculated plants and tissues were incubated in GUS assay buffer [containing 1 mM X-gluc (5-bromo-4-chloro-3-indolyl β-glucuronide) in 50 mM sodium phosphate buffer[pH 7.0, 1.0 mM potassium ferro- and ferri-cyanide, 0.01 M EDTA, pH 8.0, 0.1% Triton X100, and 20% methanol] at 37∘C for overnight. After overnight incubation, tissues were subjected bleaching using 70% ethanol for removing chlorophyll. The results were recorded after repeated washing for 4–5 times with fresh 70% ethanol solution.

### GUS Histochemical Assay for Wound and Senescence Responsiveness

To study the response of *CYP76M7* promoter to the wound and senescence, we manually induced wound on the stems as well as leaves of transgenic and control *Arabidopsis* plants using a razor blade and needle, respectively. Stems and leaves subjected to wounding were assayed for GUS expression at 10 minutes post wounding (mpw). However, for the study of senescence-responsiveness, we selected those leaves from 6 weeks-old *Arabidopsis* plants which showed natural senescence and these selected leaves were subjected to GUS histochemical assay. Three replications were used for both wound and senescence responsiveness.

### Transient GUS Assay in Rice Leaves

For transient GUS assay in rice leaves, rice cv. HR-12 was inoculated at two-leaf stage with highly virulent isolate (Moei-11) of *M. oryzae* and for mock control, plants were inoculated with 0.25% gelatin only(as discussed in the previous section). The leaves of both pathogen and mock inoculated plants were collected at 24 hpi and used for particle gun transformation. The leaves were transformed with full length as well as deletion constructs of *CYP76M7* promoter::GUS and also with promoterless pBI101 using helium driven Particle Delivery System (PDS 1000, BioRad, USA) using a standard protocol. The leaves subjected to bombardment were used for GUS assay at 48 h post bombardment.

## Results

### Expression Analysis of *CYP76M7* Gene and Identification of Transcription Start Site

**FIGURE 1:**
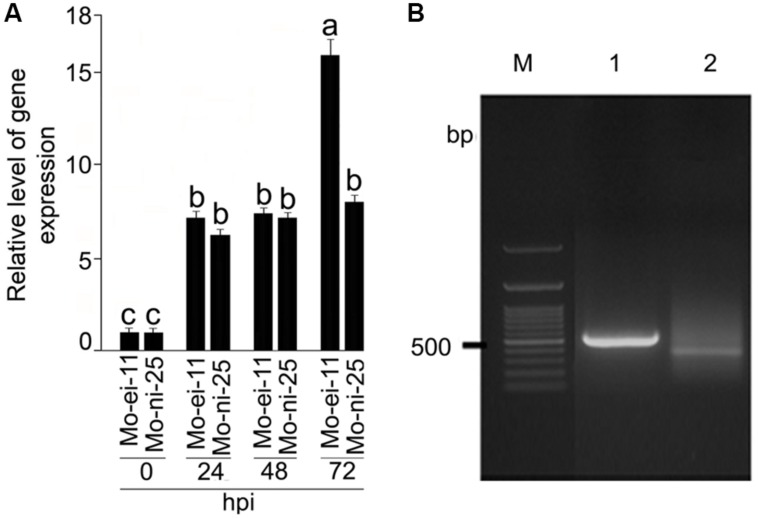
**Expression analysis *CYP76M7* transcripts and mapping of its Transcription Start Site (TSS). (A)** qRT-PCR analysis was performed to analyze the expression of *CYP76M7* in response to infection by *Magnaporthe oryzae* isolate Mo-ei-11 and Mo-ni-25 at 24, 48, and 72 hpi. Values represent mean ± SE. Means with significant deference from each other (α = 0.05) is represented with different superscripts according to Tukey’s test. **(B)** 5′ RACE (Rapid Amplification of cDNA End) PCR; M: 100 bp GeneRuler (Fermentas) 1: Positive control (provided with the kit), 2: *CYP76M7*.

Differential expression of *CYP76M7* gene between *M. oryzae* challenged and mock inoculated susceptible rice plants, HR12 (Supplementary Figure S2) at different time intervals of the experiment were recorded using RT-qPCR (**Figure 1A**). The *CYP76M7* gene was found to be 7.17, 7.39, and 15.96-fold up-regulated in rice leaf tissue after 24, 48, and 72 hpi, respectively, with *M. oryzae* isolate Mo-ei-11. Whereas, in case of *M. oryzae* isolate Mo-ni-25, a 6.24, 7.12, and 8.03-fold up-regulation was obtained. Increased upregulation of the gene was observed with an increase in the duration of infection and at 72 hpi there is a significant difference (∼7-fold) in the expression of *CYP76M7* between the rice plants inoculated with two *M. oryzae* isolates, Mo-ei-11 and Mo-ni-25. This difference in the fold change can be attributed to the highly virulent nature of Mo-ei-11 in comparison to Mo-ni-25 (Supplementary Figure S2). In order to define position of *cis*-regulatory motifs in the upstream region of *CYP76M7* gene, the TSS was determined by using 5′ RACE PCR (**Figure 1B**). The total RNA isolated from *M. oryzae* challenged rice leaves was used for RACE reaction. Analyses of the sequence obtained from cloned amplicon revealed that the TSS of the gene is defined by the nucleotide ‘A’ which was found to be located at 45 bp upstream of the translation start codon (ATG) of the *CYP76M7* gene.

### Analysis of *cis*-Regulatory Elements in *CYP76M7* Promoter

**FIGURE 2:**
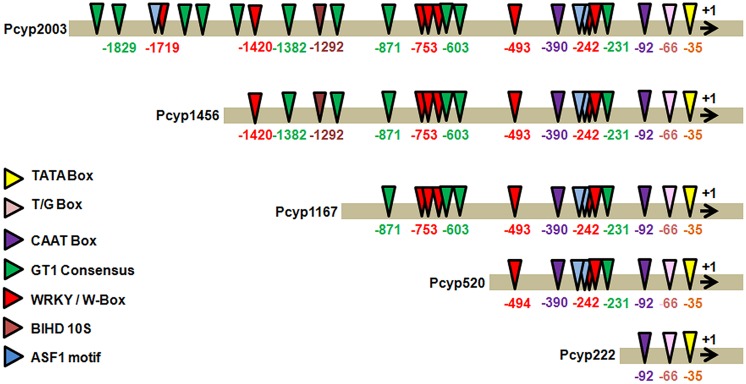
***In silico* analysis of *cis-*regulatory elements in candidate gene promoter *CYP76M7* (LOC_Os02g36110) and its four deletion fragments using PLACE online tool; Pcyp2004 (*CYP76M7* promoter-2004 bp), Pcyp1465 (*CYP76M7* promoter-1456 bp), Pcyp1167 (*CYP76M7* promoter-1167 bp), Pcyp520 (*CYP76M7* promoter-520 bp), and Pcyp222 (*CYP76M7* promoter-222 bp)**.

The 2,004 bp 5′ regulatory region of the promoter of *CYP76M7*gene was analyzed for the presence of *cis*-regulatory elements, found to be associated with pathogen inducible promoters using NEW PLACE database (**Figure 2**). The important core promoter elements such as TATA Box (CTATAAATAC) at 35 bp upstream to TSS and CAAT box sequences at 92 and 390 bp upstream to TSS were obtained. We also found the presence of motifs like T/G-box (AACGTG) at -66 bp site; while 11 boxes of GT-1 were present at -1848, -1829, 1681, -1518, -1480, -1,382, -1126, -871, -714, -603, and -231 bp positions. An ASF1 motif which is characterized as basic Leucine Zipper (bZIP) protein binding site and reported to be involved in salicylic acid (SA) induced expression of genes (Hwang and Hwang, 2010) was found at -221, -242, and -1720 bps. In addition to this, eight ‘W’ boxes (TTGAC) containing elements were present at -1,719, -1,420, -753, -669, -621, -493, -250, and -242 bps positions. A BIHD1OS, the binding site of *OsBIHD1*, a rice BELL homeodomain transcription factor, was found at -1292 bp .

### Establishment of Infection of *Arabidopsis* with *M. oryzae*

**FIGURE 3:**
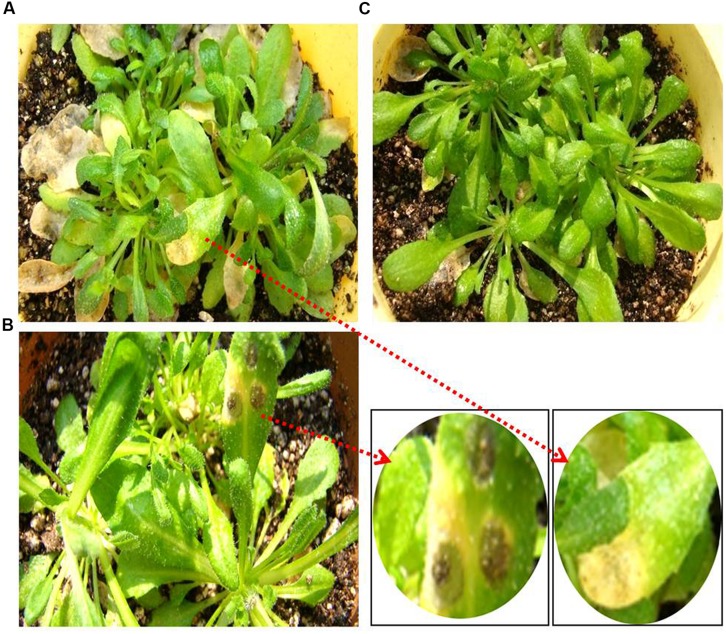
**Phenotypic response of *Arabidopsis* plant inoculated with *M. oryzae* isolate Mo-ei-11**. Three days after **(A)** spray inoculation and **(B)** spot inoculation with *M. oryzae*; **(C)** 3 days after mock inoculation with 0.2% gelatin.

The phenotypic response of *Arabidopsis* (ecotype Col-0) plants inoculated by both spray and spot methods with a spore suspension of *M. oryzae* was observed at 3 dpi. At the site of spot inoculation, typical necrotic spots with yellow halo were observed in the case of *M. oryzae* challenged plants, whereas no symptoms were found in mock inoculated plants (**Figure 3**). This experiment was performed in five replications to establish proper screening protocol. With spray inoculation the pathogen infected leaves developed yellow necrotic patches while no changes were observed in mock inoculated plants.

### Validation of the Promoter for Pathogen Responsiveness

**FIGURE 4:**
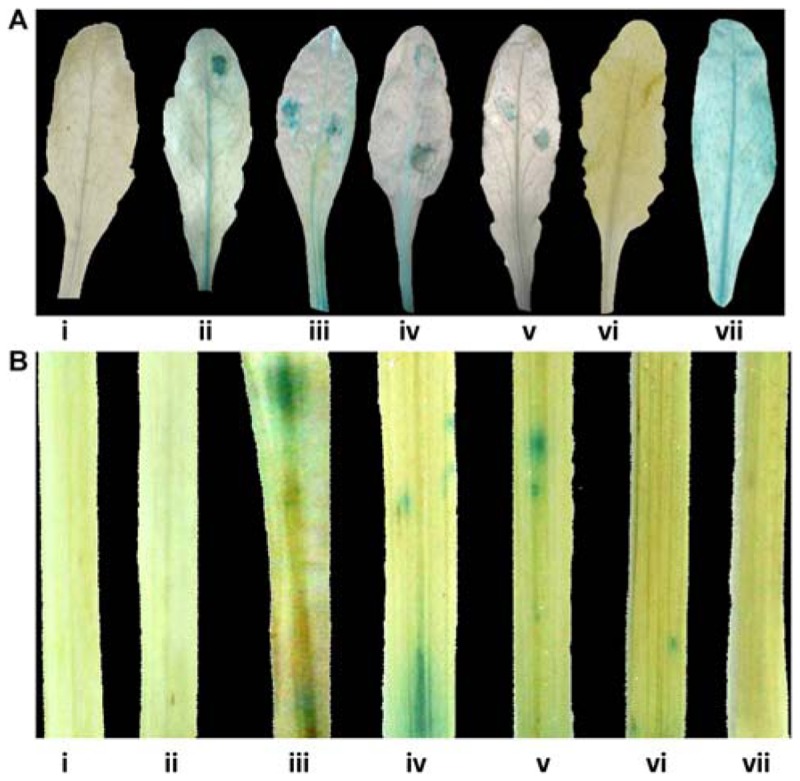
**Glucuronidase (GUS) histochemical assays for functional validation of different *CYP76M7* promoter deletion. (A)** *Arabidopsis* mock inoculated (i) Pcyp2004; and *M. oryzae* challenged (ii) Pcyp2004; (iii) Pcyp1456; (iv) Pcyp1167; (v) Pcyp520; (vi) Pcyp222; (vii) constitutive 35 S promoter (positive control) plants. **(B)** Transient expression in rice leaves bombarded with (i) pBI 101 empty vector; (ii) mock inoculated Pcyp2004; and *M. oryzae* challenged (iii) Pcyp2004; (iv) Pcyp1456; (v) Pcyp1167; (vi) Pcyp520: (vii) Pcyp222.

Pathogen responsiveness of *CYP76M7* promoter was validated by GUS histochemical assay in stable and transiently transformed *Arabidopsis* and rice plants, respectively. Independent transgenic *Arabidopsis* plants with four promoter deletion fragments consisting of Pcyp2004 (2004 bp), Pcyp1456 (1456 bp), Pcyp1167 (1167 bp), and Pcyp520 (520 bp) regions could express GUS in response to pathogen infection at 24 hpi, whereas no such induction was observed in the mock inoculated plants (**Figure 4A**; Supplementary Figure S3A). However, no induction of GUS gene was observed in both pathogen-challenged and mock-inoculated transgenic plants containing Pcyp222 (222 bp) promoter fragment. These findings were further validated by transient expression of GUS in rice leaves. The results obtained in the case of rice were similar to those of *Arabidopsis* plants. The promoter deletions Pcyp2004, Pcyp1456, Pcyp1167, and Pcyp520 were transiently induced by *M. oryzae* infection (**Figure 4B**; Supplementary Figure S3B).

### Validation for Wound and Senescence Responsiveness

**FIGURE 5:**
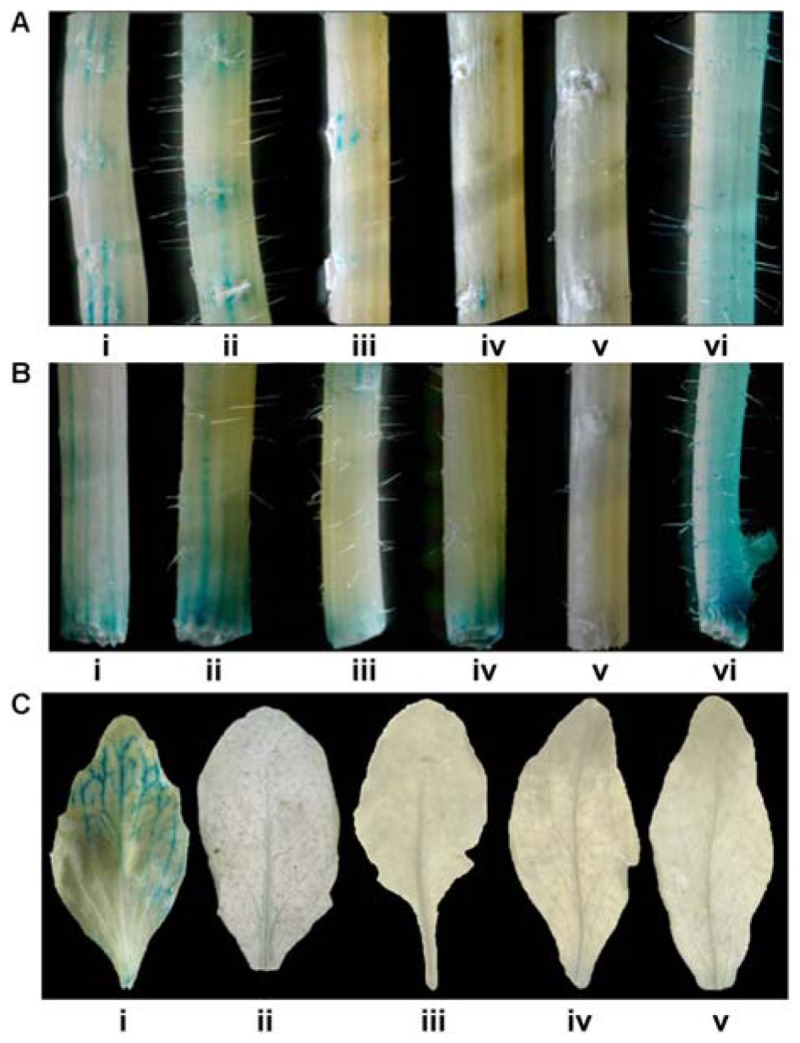
**Glucuronidase histochemical assay to know wound responsiveness of *CYP76M7* promoter**. Stems of different *Arabidopsis* plants with **(A)** surface wounding and **(B)** wounding at the cut ends of (i) Pcyp2004; (ii) Pcyp1456; (iii) Pcyp1167; (vi) Pcyp520: (v) Pcyp222; (vi) 35S promoter. **(C)** GUS histochemical assay for the leaves undergoing senescence in different transgenic *Arabidopsis* plants containing CYP76M7 deletions; (i) Pcyp2004; (ii) Pcyp1456; (iii) Pcyp1167; (iv) Pcyp520: (v) Pcyp222.

The Responsiveness of *CYP76M7* promoter to wound as well as senescence was validated by GUS histochemical assay of transformed *Arabidopsis* plants. The results obtained revealed that transgenic *Arabidopsis* plants with promoter deletion fragments Pcyp2004, Pcyp1456, Pcyp1167, and Pcyp520 could express GUS in response to pathogen infection at 10 mpw but such expression was limited to cut ends of the stem as well as wounded stem surfaces (**Figures 5A,B**). Further, the responsiveness of *CYP76M7* promoter to senescence revealed that *Arabidopsis* plants with only full length promoter Pcyp2004 could drive the expression of GUS transcript and no GUS induction was observed in transgenic *Arabidopsis* plants with deletion fragments Pcyp1456, Pcyp1167, Pcyp520, and Pcyp222 (**Figure 5C**).

## Discussion

Use of spatially and temporally inducible promoters is considered as an important in obtaining high degree of resistance to plant pathogen. So far only a few plant pathogen-inducible promoters have been well characterized, with respect to their specific patterns and regulation of expression (Gurr and Rushton, 2005). There is a strong need for the identification of early and race-non-specific pathogen-inducible promoters, preferably those which could work across pathogen and species-specific barriers. The tobacco *hsr203J* promoter is an ideal candidate being rapidly induced by more than one pathogen, as early as 9 hpi (Pontier et al., 2001). In the present investigation strong induction of *CYP76M7* gene was obtained in the susceptible rice cultivar HR-12 from 24 to 72 hpi in response to infection with geographically diverse isolates of *M. oryzae*. An increased upregulation of the gene was observed with an increase in the duration of infection and at 72 hpi there is a significant difference (∼7-fold) in the expression of *CYP76M7* between the rice plants inoculated with two *M. oryzae* isolates, Mo-ei-11, and Mo-ni-25. The sevenfold difference in the expression pattern of *CYP76M7* could be attributed to the highly virulent nature of Mo-ei-11 in comparison to Mo-ni-25. Similar results of 8.05, 8.08, and 10.45-fold change were obtained at 24, 48, and 72 hpi, respectively, for *CYP76M7* gene using microarray experiments (Unpublished data). Thus, the expression pattern of the *CYP76M7* correlates well by both the approaches. Previous study has also reported the induction of *CYP76M7* in resistant and susceptible rice genotypes (Bagnaresi et al., 2012).

In the present study, nucleotide sequence analysis of *CYP76M7* promoter from *O. sativa* cv. HR12 showed the presence of TATA box, CAAT box, T/G, GT-1, and ASF1 motifs, as well as WRKY TF and BIHD1OS binding sites. TATA box is necessary for binding of general transcription factors, whereas CAAT box is responsible for the tissue specific promoter activity (Shirsat et al., 1989). T/G-box (AACGTG) has the binding site of basic region/helix–loop–helix (bHLH) proteins and is known to be involved in Jasmonic Acid (JA) induction (Boter et al., 2004). The GT-1 motif (GAAAAA) is found to have a role in pathogen and salt-induced gene expression in soybean (Park et al., 2004). The ASF1 motif which is characterized as bZIP protein binding site has been reported to be involved in SA induced expression of the genes (Hwang and Hwang, 2010). Presence of eight W-boxes (TTGAC) containing elements, which are known to bind the WRKY family of transcription factors (Rushton and Somssich, 1998; Nischiuchi et al., 2004) involved in SA -induced expression signifies their role in responsiveness of *CYP76M7* promoter to *M. oryzae* infection. The W-box elements found in the *CYP76M7* promoter were found to have a WRKY71OS TF binding site and these elements are reported to be induced by rice blast fungus *M. oryzae* (Kim et al., 2000; Liu et al., 2006). BIHD1OS sites are the binding site of *OsBIHD1*, rice BELL homeodomain transcription found in disease resistance response genes (Luo et al., 2005).

Previously, the pathogenecity of *M. oryzae* on *Arabidopsis* has been reported (Park et al., 2009). Our assumption in carrying out the functional validation of *CYP76M7* promoter in *Arabidopsis* were the following (i) to determine if, the promoter would be functional across monocot-dicot barrier and (ii) to understand the nuances of pathogen-responsive expression in a non-host model plant system.

The PmPR10-1.13 promoter from *Pinus monticola*, induced by *Cronartium ribicola* was reported to be directing the enhanced expression of GUS expression following pathogen infection and by wounding treatment in case of transgenic *Arabidopsis* plants (Liu et al., 2005). Similarly, promoter of the soybean *GmaPPO12* gene was found to be rapidly and strongly induced by *Phytophthora sojae* infection and induced *GUS* reporter gene expression after infection in both transient expression assays in *Nicotiana benthamiana* and stable transgenic soybean hairy roots (Chai et al., 2013). In the present study, 5′ deletions of *CYP76M7* promoter were fused with promoter less *GUS* reporter gene and its expression was analyzed in stable and transiently expressed *Arabidopsis* and rice systems, respectively. In *Arabidopsis*, the *CYP76M7* promoter-directed the expression of *GUS* gene upon pathogen infection and also by wounding treatment and senescence, both of which generally mimic the endogenous expression pattern of pathogen responsive genes (Liu et al., 2005). Further the transient expression analysis in tomato reports that wounding induces several resistance genes (*PINIIb*, *PR1b*, *PR5*, *PR7,* and peroxidase) locally and/or systemically and ethylene perception is needed for the full scale induction of all these genes, except peroxidase (Francia et al., 2008). Our study indicates that the promoter which is responsive to pathogen might also be induced by wounding. But, the GUS expression in *Arabidopsis* was restricted to only stem and petioles and no expression was observed in wounded leaves. Our study also reveals that the promoter region spanning -222 to -520 bp confers *M. oryzae* as well as wound inducibility to *CYP76M7* promoter, which may be attributed to the presence of a cluster of functional motifs like three W-boxes, two ASF1 motifs and one GT-1 element in the corresponding region. These motifs have been functionally validated and are known to impart stress inducible response to the respective promoters (Rushton and Somssich, 1998; Nischiuchi et al., 2004; Park et al., 2004; Zhang et al., 2007). On the contrary, the response of *CYP76M7* to senescence revealed that only full length promoter could induce the expression of GUS gene. This may be attributed to the presence of the ASF-1 motif at -1720 position. Previously, it has been shown that anti*s*ilencing function *1a* (*ASF1a*), plays an important role in the formation of SAHF, which contributes to the irreversible exit from the cell cycle in the cells undergoing senescence by inhibiting the expression of cell proliferation-promoting genes (Zhang et al., 2007).

In this study, we have cloned, characterized and functionally validated the *CYP76M7* gene promoter for its induction during *M. oryzae* infection in *Arabidopsis* and rice. The pathogenicity of *M. oryzae* on *Arabidopsis* was confirmed multiple times. GUS histochemical assay analysis of stable transformed *Arabidopsis* as well as the transient transformed rice leaves revealed that the promoter of *CYP76M7* is induced by *M. oryzae*. Therefore, we report that the cluster of two W-boxes, two ASF1 motifs and a single GT-1 element present between -222 to -520 bp may contribute to the *M. oryzae* inducible nature of *CYP76M7* promoter. Therefore the promoter cloned and functionally validated in this study would be an ideal candidate for a *M. oryzae* inducible promoter.

## Author Contributions

TRS: conceived and designed the experiments. JV and DN: performed the experiments and analyzed the data. TRS, JV, and DN: wrote the paper.

## Acknowledgments

The financial assistance received by TRS from National Agricultural Innovation Project (NAIP; C4/C1071), Indian Council of Agricultural Research, is gratefully acknowledged. TRS is also thankful to the head of the National Phytotron Facility for providing space for plant growth and maintenance. We thank Dr. M. Variar and Dr. U. D. Singh for providing *M. oryzae* isolates from their respective regions.

## Supplementary material

The Supplementary Material for this article can be found online at: http://journal.frontiersin.org/article/10.3389/fpls.2015.00371/abstract

## Footnotes

1 http://www.quantprime.de/

2 http://www.phrap.org/

3 www.softberry.com

4 http://www.dna.affrc.go.jp/PLACE/

## References

[B1] Bagnaresi P. Biselli C. Orru L. Urso S. Crispino L. Abbruscato P. (2012). Comparative transcriptome profiling of the early response to *Magnaporthe oryzae* in durable resistant vs susceptible rice (*Oryza sativa* L.) genotypes. *PLoS ONE* 7:e51609 10.1371/journal.pone.0051609 PMC3520944 23251593

[B2] Boter M. Ruiz-Rivero O. Abdeen A. Prat S. (2004). Conserved MYC transcription factors play a key role in jasmonate signaling both in tomato and *Arabidopsis*. *Genes Dev.* 18 1577–1591. 10.1101/gad.297704 15231736 PMC443520

[B3] Chai C. Lin Y. Shen D. Wu Y. Li H. Dou D. (2013). Identification and functional characterization of the soybean gmappo12 promoter conferring *Phytophthora sojae* induced expression. *PLoS ONE* 8:e67670 10.1371/journal.pone.0067670 PMC3695865 23840763

[B4] Das A. Soubam D. Singh P. K. Thakur S. Singh N. K. Sharma T. R. (2012). A novel blast resistance gene, Pi54rh cloned from wild species of rice, *Oryza rhizomatis* confers broad spectrum resistance to *Magnaporthe oryzae*. *Funct. Integr. Genomics* 12 215–228. 10.1007/s10142-012-0284-1 22592658

[B5] De Wit P. J. G. M. (1992). Molecular characterisation of gene-for-gene systems in plant-fungus interactions and the application of avirulence genes in control of plant pathogens. *Annu. Rev. Phytopathol.* 30 391–418. 10.1146/annurev.py.30.090192.002135 18647100

[B6] Devanna N. B. Vijayan J. Sharma T. R. (2014). The blast resistance gene Pi54of cloned from *Oryza officinalis* interacts with Avr-Pi54 through its novel non-LRR domains. *PLoS ONE* 9:e104840 10.1371/journal.pone.0104840 PMC4128725 25111047

[B7] Eulgem T. Rushton P. J. Robatzek S. Somssich I. E. (2000). The WRKY superfamily of plant transcription factors. *Trends Plant Sci.* 5 199–206. 10.1016/S1360-1385(00)01600-9 10785665

[B8] Ewing B. Green P. (1998). Base calling sequencer traces using Phred II. Error probabilities. *Genome Res.* 8 186–194. 10.1101/gr.8.3.175 9521922

[B9] Francia D. Demaria D. Calderini O. Ferraris L. Valentino D. Arcioni S. (2008). Do pathogen-specific defense mechanisms contribute to wound-induced resistance in tomato? *Plant Signal. Behav.* 3 340–341. 10.4161/psb.3.5.5351 19841665 PMC2634277

[B10] Gurr S. J. Rushton P. J. (2005). Engineering plants with increased disease resistance: how are we going to express it? *TRENDS Biotech.* 23 283–290. 10.1016/j.tibtech.2005.04.009 15922080

[B11] Hammond-Kosack K. E. Parker J. E. (2003). Deciphering plant pathogen communication: fresh perspectives for molecular resistance breeding. *Curr. Opin. Biotechnol.* 14 77–193. 10.1016/S0958-1669(03)00035-1 12732319

[B12] Higo K. Ugawa Y. Iwamoto M. Korenaga T. (1999). Plant cis-acting regulatory DNA elements (PLACE) database. *Nucl. Acids Res.* 27 297–300. 10.1093/nar/27.1.297 9847208 PMC148163

[B13] Hulbert S. H. Webb C. A. Smith S. M. Sun Q. (2001). Resistance gene complexes: evolution and utilization. *Annu. Rev. Phytopathol* 39 285–312. 10.1146/annurev.phyto.39.1.285 11701867

[B14] Hwang S. H. Hwang D. J. (2010). Isolation and characterization of rice NPR1 promoter. *Plant Biotechnol. Rep.* 4 29–35. 10.1007/s11816-009-0116-5

[B15] Kim C. Y. Lee S. H. Park H. C. Bae C. G. Cheong Y. H. Choi Y. J. (2000). Identification of rice blast fungal elicitor-responsive genes by differential display analysis. *Mol. Plant Microbe. In*. 13 470–474. 10.1094/MPMI.2000.13.4.470 10755311

[B16] Koga J. Shimura M. Oshima K. Ogawa N. Yamauchi T. Ogasawara N. (1995). Phytocassanes A, B, C, and D, novel diterpene phytoalexins from rice, *Oryza sativa* L. *Tetrahedron* 51 7907–7918. 10.1016/0040-4020(95)00423-6

[B17] Liu J. J. Ekramoddoullah A. K. Piggott N. Zamani A. (2005). Molecular cloning of a pathogen/wound inducible PR10 promoter from *Pinus monticola* and characterization in transgenic *Arabidopsis* plants. *Planta* 221 159–169. 10.1007/s00425-004-1428-x 15609047

[B18] Liu X. Bai X. Wang X. Chu C. (2006). OsWRKY71, a rice transcription factor, is involved in rice defense response. *J. Plant Physiol.* 164 969–979. 10.1016/j.jplph.2006.07.006 16919842

[B19] Livak K. J. Schmittgen T. D. (2001). Analysis of relative gene expression data using real-time quantitative PCR and the 2(-Delta Delta C(T) method. *Methods* 25 402–408. 10.1006/meth.2001.1262 11846609

[B20] Luo H. Song F. Goodman R. M. Zheng Z. (2005). Up-regulation of OsBIHD1, a rice gene encoding BELL homeodomain transcriptional factor, in disease resistance responses. *Plant Biol.* 7 459–468. 10.1055/s-2005-865851 16163610

[B21] Maeda K. Houjyou Y. Komatsu T. Hori H. Kodaira T. Ishikawa A. (2009). AGB1 and PMR5 contribute to PEN2-mediated preinvasion resistance to *Magnaporthe oryzae* in *Arabidopsis thaliana*. *Mol. Plant Microbe. Interact.* 22 1331–1340. 10.1094/MPMI-22-11-1331 19810803

[B22] Maeda K. Houjyou Y. Komatsu T. Hori H. Kodaira T. Ishikawa A. (2010). Nonhost resistance to *Magnaporthe oryzae* in *Arabidopsis thaliana*. *Plant Signal. Behav.* 5 755–756. 10.4161/psb.5.6.11770 20404515 PMC3001581

[B23] Murashige T. Skoog F. (1962). A revised medium for rapid growth and bioassays with tobacco cultures. *Physiol. Plant.* 15 473–497. 10.1111/j.1399-3054.1962.tb08052.x

[B24] Nakao M. Nakamura R. Kita K. Inukai R. Ishikawa A. (2011). Non-host resistance to penetration and hyphal growth of *Magnaporthe oryzae* in *Arabidopsis*. *Sci. Rep.* 10.1038/srep00171 PMC3240950 22355686

[B25] Nischiuchi T. Shinshi H. Suzuki K. (2004). Rapid and transient activation of transcription of the ERF3 gene by wounding in tobacco leaves – Possible involvement of NtWRKYs and autorepression. *J. Biol. Chem.* 279 55355–55361. 10.1074/jbc.M409674200 15509567

[B26] Ohme-Tagaki M. Suzuki K. Shinshi H. (2000). Regulation of ethylene-induced transcription of defense genes. *Plant Cell Physiol.* 41 1187–1192. 10.1093/pcp/pcd057 11092902

[B27] Okada A. Shimizu T. Okada K. Kuzuyama T. Koga J. Shibuya N. (2007). Elicitor induced activation of the methylerythritol phosphate pathway towards phytoalexin biosynthesis in rice. *Plant Mol. Biol.* 65 177–187. 10.1007/s11103-007-9207-2 17634747

[B28] Park H. C. Kim M. L. Kang Y. H. Jeon J. M. Yoo J. H. Kim M. C. (2004). Pathogen- and NaCl induced expression of the SCaM-4 promoter is mediated in part by a GT-1 box that interacts with a GT-1-like transcription factor. *Plant Physiol.* 135 2150–2161. 10.1104/pp.104.041442 15310827 PMC520786

[B29] Park J. Y. Jin J. Lee Y. W. Kang S. Lee Y. H. (2009). Rice blast fungus (*Magnaporthe oryzae*) infects Arabidopsis via a mechanism distinct from that required for the infection of rice. *Plant Physiol.* 149 474–486. 10.1104/pp.108.129536 18987215 PMC2613700

[B30] Pontier D. Balague C. Bezombes-Marion I. Tronchet M. Deslandes L. Roby D. (2001). Identification of a novel pathogen-responsive element in the promoter of the tobacco gene HSR203J, a molecular marker of the hypersensitive response. *Plant J.* 26 495–507. 10.1046/j.1365-313x.2001.01049.x 11439136

[B31] RoyChowdhury M. Jia Y. L. Jackson A. Jia M. H. Fjellstrom R. Cartwright R. D. (2012). Analysis of rice blast resistance gene Pi-z in rice germplasm using pathogenicity assays and DNA markers. *Euphytica* 184 35–46. 10.1007/s10681-011-0481-3

[B32] Rushton P. J. Somssich I. E. (1998). Transcriptional control of plant genes responsive to pathogens. *Curr. Opin. Plant Biol.* 1 311–315. 10.1016/1369-5266(88)80052-9 10066598

[B33] Rushton P. J. Torres J. T. Parniske M. Wernert P. Hahlbrock K. Somssich I. E. (1996). Interaction of elicitor-induced DNA binding proteins with elicitor response elements in the promoters of parsley PR1 genes. *EMBO J.* 15 5690–5700. 8896462 PMC452313

[B34] Sharma T. R. Rai A. K. Gupta S. K. Vijayan J. Devanna B. N. Ray S. (2012). Rice blast management through host resistance: retrospect and prospects. *Agric. Res.* 1 37–52. 10.1007/s40003-011-0003-5

[B35] Shirsat A. Wilford N. Croy R. Boulter D. (1989). Sequences responsible for the tissue specific promoter activity of a pea legumin gene in tobacco. *Mol. Gen. Genet.* 215 326–331. 10.1007/BF00339737 2710102

[B36] Swaminathan S. Moron D. Wang Q. Fulton D. B. Peters R. J. (2009). CYP76M7 Is an ent-cassadiene C11a-hydroxylase defining a second multifunctional diterpenoid biosynthetic gene cluster in rice. *Plant Cell* 21 3315–3325. 10.1105/tpc.108.063677 19825834 PMC2782285

[B37] Zhang R. Chen W. Adams P. D. (2007). Molecular dissection of formation of senescence-associated heterochromatin foci. *Mol. Cell Biol.* 27 2343–2358. 10.1128/MCB.02019-06 17242207 PMC1820509

