# Corrigendum: Cloning and functional validation of early inducible *Magnaporthe oryzae* responsive *CYP76M7* promoter from rice

**DOI:** 10.3389/fpls.2018.00939

**Published:** 2018-06-27

**Authors:** Joshitha Vijayan, B. N. Devanna, Nagendra K. Singh, Tilak R. Sharma

**Affiliations:** National Research Centre on Plant Biotechnology, New Delhi, India

**Keywords:** *Arabidopsis*, *CYP76M7*, GUS, *Magnaporthe*, promoter analysis, rice

In the original article, there was a mistake in (Figure 4) as published (Similar morphology of leaves in 4Ai and 4Avi). The corrected (Figure 4) appears below. In the original article, there was a mistake in (Figure 5) as published (Similar morphology of the leaves in 5Ciii and 5Cv). The corrected (Figure 5) appears below.

**Figure 4:**
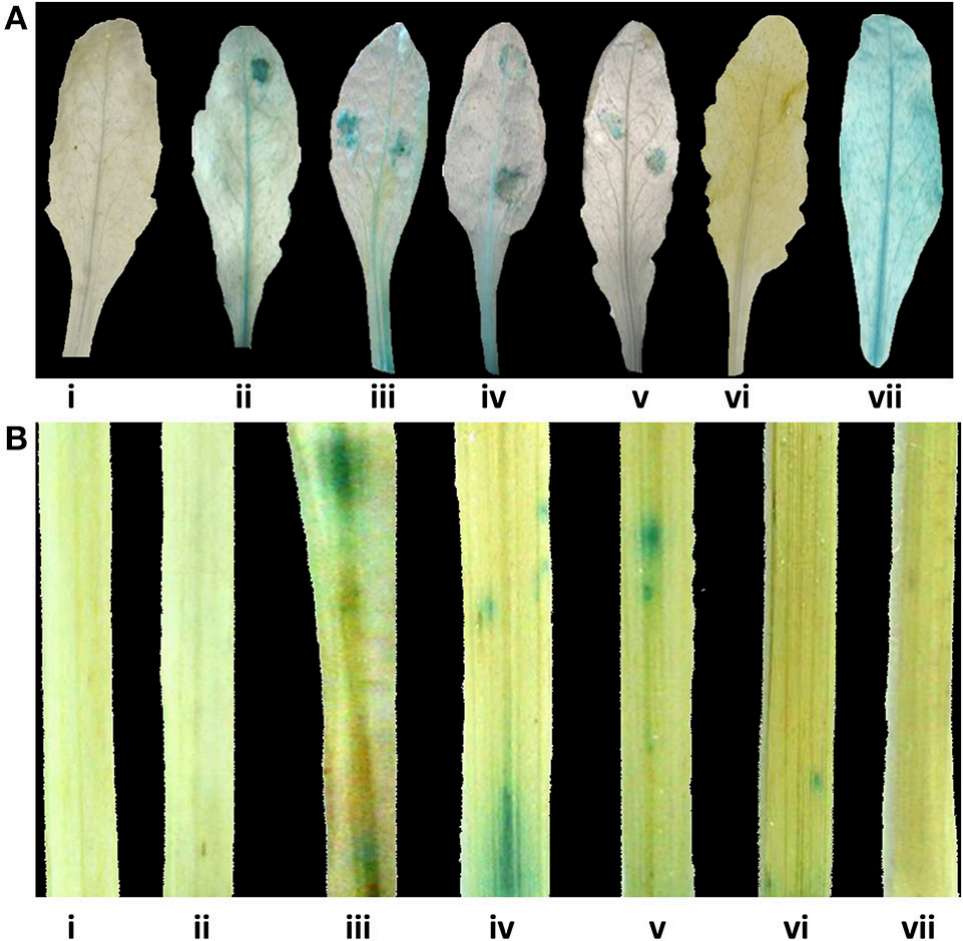
Glucuronidase (GUS) histochemical assays for functional validation of different *CYP76M7* promoter deletion. **(A)** *Arabidopsis* mock inoculated (i) Pcyp2004; and *M. oryzae* challenged (ii) Pcyp2004; (iii) Pcyp1456; (iv) Pcyp1167; (v) Pcyp520; (vi) Pcyp222; (vii) constitutive 35 S promoter (positive control) plants. **(B)** Transient expression in rice leaves bombarded with (i) pBI 101 empty vector; (ii) mock inoculated Pcyp2004; and *M. oryzae* challenged (iii) Pcyp2004; (iv) Pcyp1456; (v) Pcyp1167; (vi) Pcyp520: (vii) Pcyp222.

**Figure 5:**
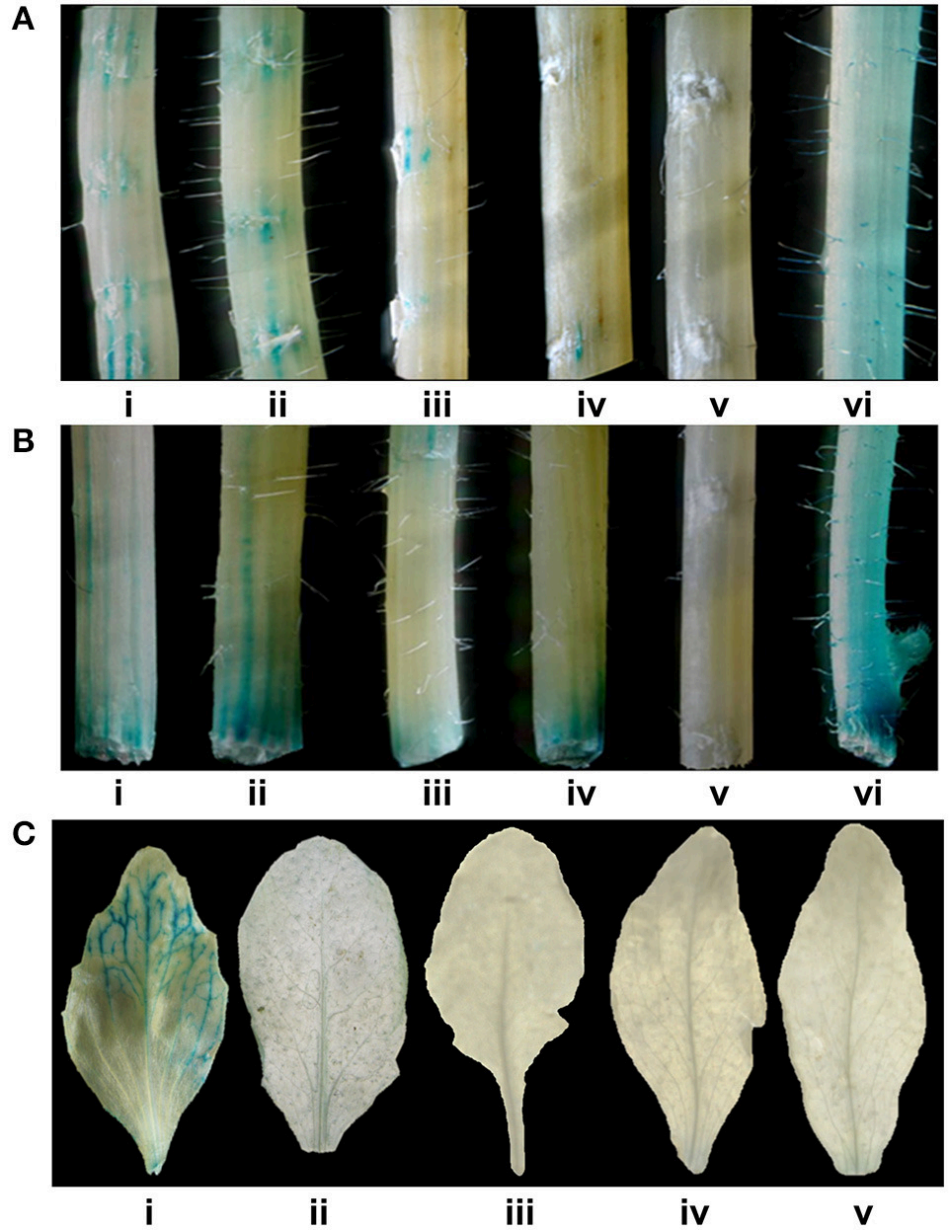
Glucuronidase histochemical assay to know wound responsiveness of *CYP76M7*promoter. Stems of different *Arabidopsis* plants with**(A)** surface wounding and **(B)** wounding at the cut ends of (i) Pcyp2004; (ii) Pcyp1456; (iii) Pcyp1167; (vi) Pcyp520: (v) Pcyp222; (vi) 35S promoter. **(C)** GUS histochemical assay for the leaves undergoing senescence in different transgenic *Arabidopsis* plants containing CYP76M7 deletions; (i) Pcyp2004; (ii) Pcyp1456; (iii) Pcyp1167; (iv) Pcyp520: (v) Pcyp222.

It is further clarified that the authors reviewed and confirmed the integrity of the original data. Once integrity of these archived data was established, replicate images of leaves from the original samples were selected corresponding to the experimental set-up reported in the manuscript.

The authors apologize for these error and state that this does not change the scientific conclusions of the article in any way.

The original article has been updated.

## Conflict of interest statement

The authors declare that the research was conducted in the absence of any commercial or financial relationships that could be construed as a potential conflict of interest.

