# Supplementary figures and images for: Cloning and functional validation of early inducible Magnaporthe oryzae responsive CYP76M7 promoter from rice
Source: Front Plant Sci. 2015 May 22;6:371. doi: 10.3389/fpls.2015.00371 (PMC4441127; doi:10.3389/fpls.2015.00371)

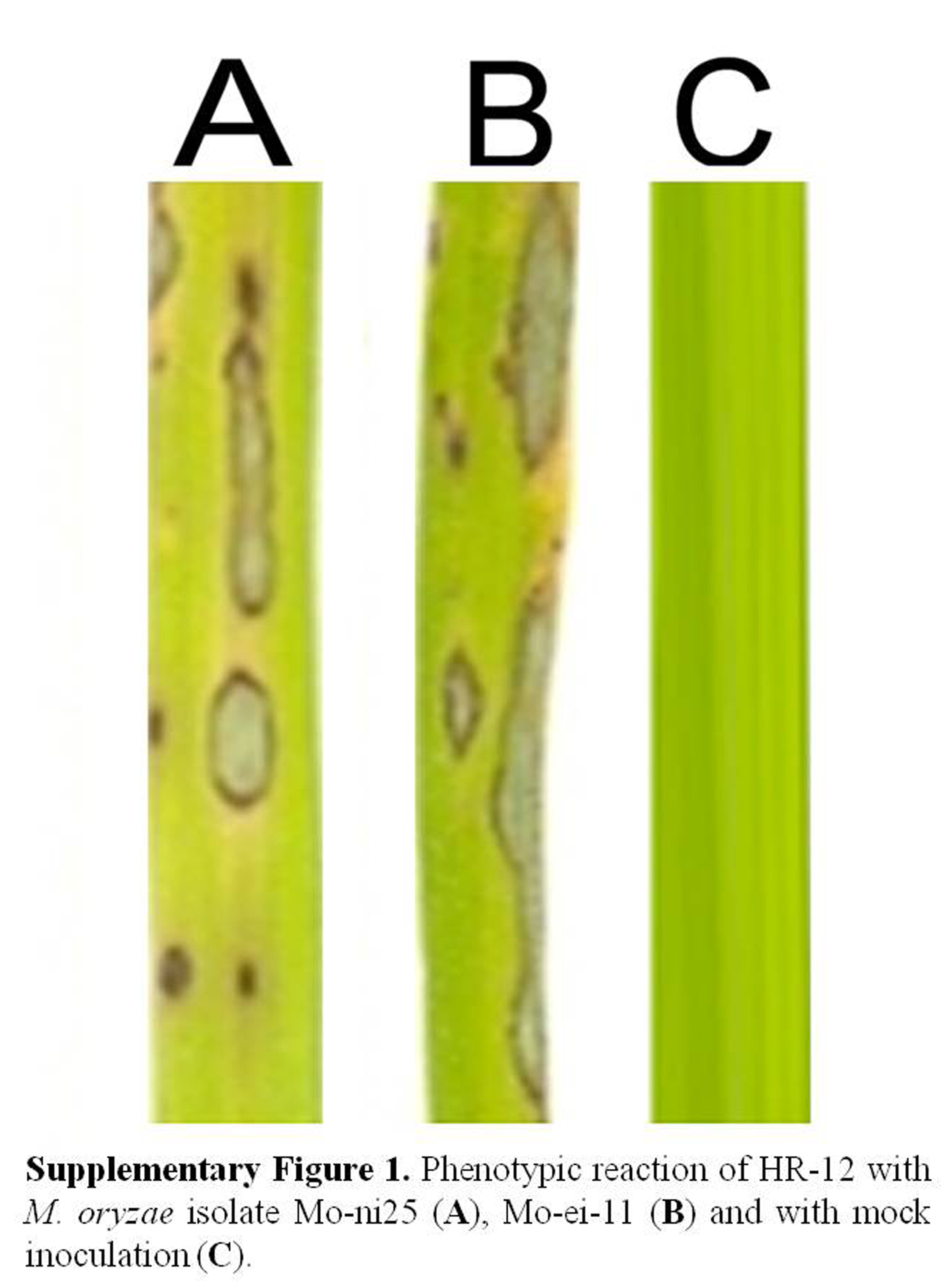

Supplement: Supplementary file 1 [file Image_1.TIF]

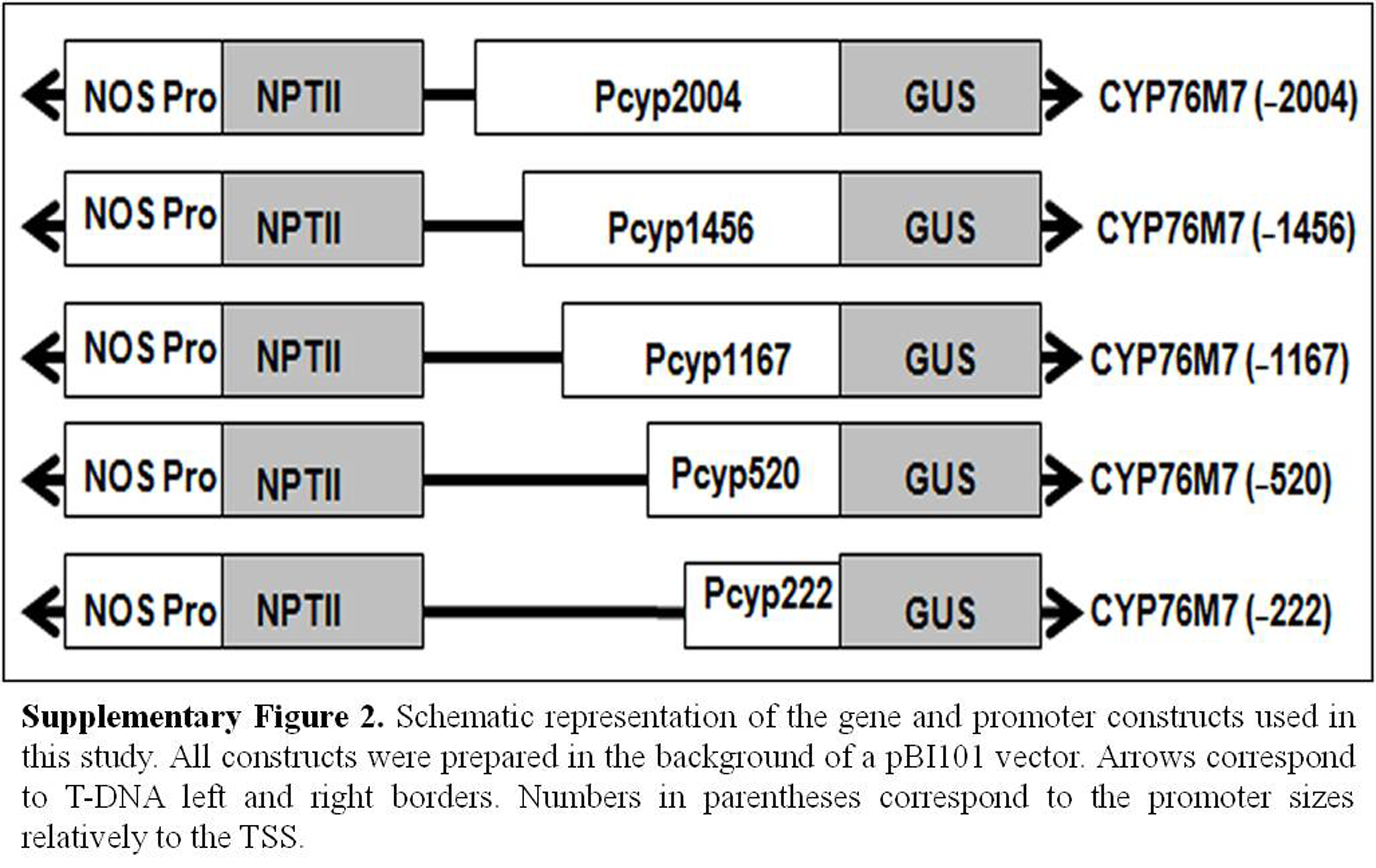

Supplement: Supplementary file 2 [file Image_2.TIF]

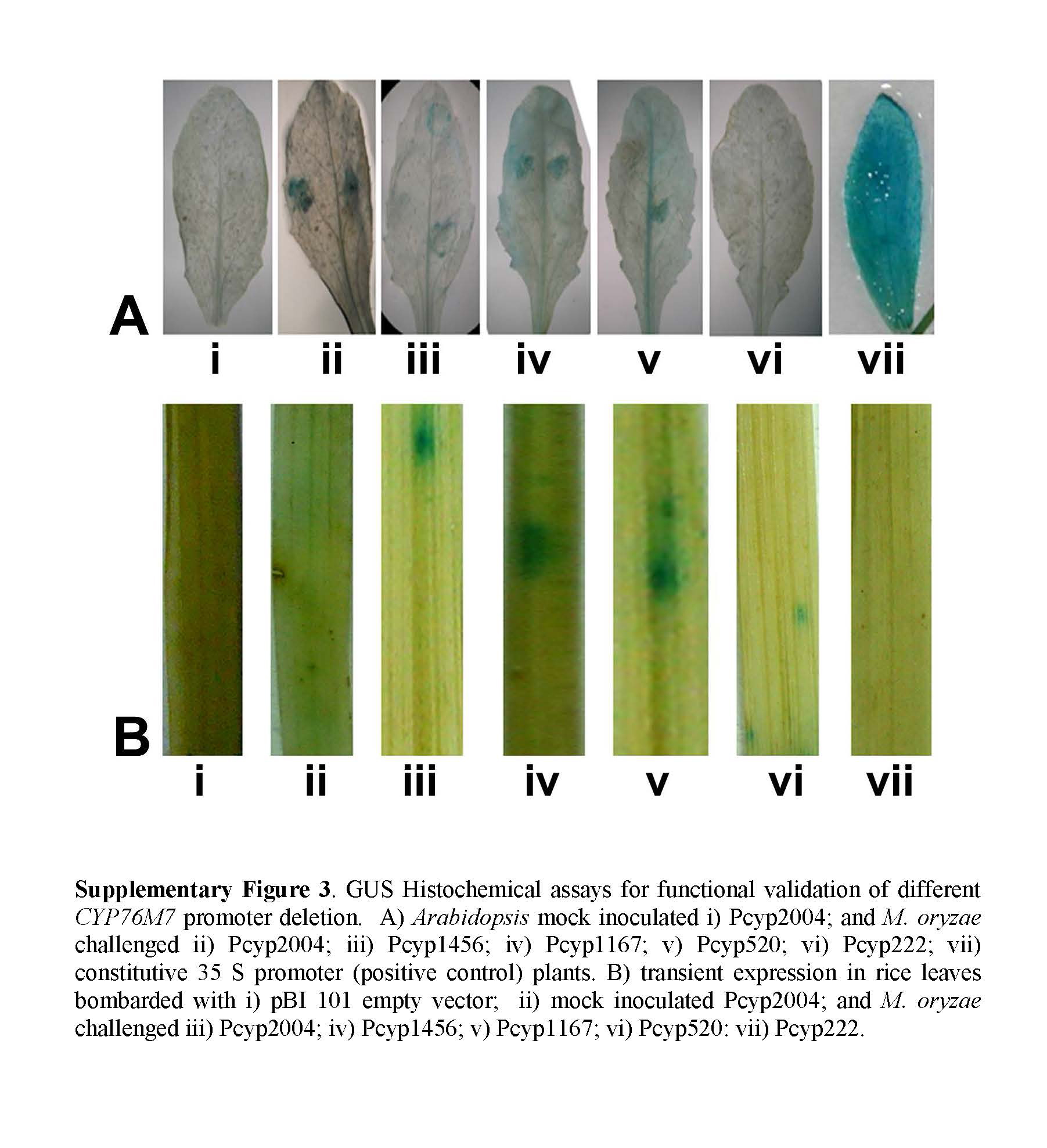

Supplement: Supplementary file 3 [file Image_3.JPEG]
